# *RhWRKY33* Positively Regulates Onset of Floral Senescence by Responding to Wounding- and Ethylene-Signaling in Rose Plants

**DOI:** 10.3389/fpls.2021.726797

**Published:** 2021-11-05

**Authors:** Weikun Jing, Qingcui Zhao, Shuai Zhang, Daxing Zeng, Jiehua Xu, Hougao Zhou, Fenglan Wang, Yang Liu, Yonghong Li

**Affiliations:** ^1^School of Food and Drug, Shenzhen Polytechnic, Shenzhen, China; ^2^Postdoctoral Innovation Practice Base, Shenzhen Polytechnic, Shenzhen, China; ^3^School of Construction Engineering, Shenzhen Polytechnic, Shenzhen, China; ^4^College of Horticulture and Landscape Architecture, Zhongkai University of Agriculture and Engineering, Guangzhou, China

**Keywords:** rose, senescence, wound, ethylene, *RhWRKY33*

## Abstract

Rose plants are one of the most important horticultural crops, whose commercial value mainly depends on long-distance transportation, and wounding and ethylene are the main factors leading to their quality decline and accelerated senescence in the process. However, underlying molecular mechanisms of crosstalk between wounding and ethylene in the regulation of flower senescence remain poorly understood. In relation to this, transcriptome analysis was performed on rose flowers subjected to various treatments, including control, wounding, ethylene, and wounding- and ethylene- (EW) dual treatment. A large number of differentially expressed genes (DEGs) were identified, ranging from 2,442 between the ethylene- and control-treated groups to 4,055 between the EW- and control-treated groups. Using weighted gene co-expression network analysis (WGCNA), we identified a hub gene *RhWRKY33* (rchiobhmchr5g0071811), accumulated in the nucleus, where it may function as a transcription factor. Moreover, quantitative reverse transcription PCR (RT-qPCR) results showed that the expression of *RhWRKY33* was higher in the wounding-, ethylene, and EW-treated petals than in the control-treated petals. We also functionally characterized the *RhWRKY33* gene through virus-induced gene silencing (VIGS). The silencing of *RhWRKY33* significantly delayed the senescence process in the different treatments (control, wounding, ethylene, and EW). Meanwhile, we found that the effect of *RhWRKY33*-silenced petals under ethylene and EW dual-treatment were stronger than those under wounding treatment in delaying the petal senescence process, implying that *RhWRKY33* is closely involved with ethylene and wounding mediated petal senescence. Overall, the results indicate that *RhWRKY33* positively regulates the onset of floral senescence mediated by both ethylene and wounding signaling, but relies heavily on ethylene signaling.

## Introduction

Rose plants are one of the most important horticultural crops worldwide; their ornamental and economic value mainly depends on the full expansion and slow senescence of their petals (Lu et al., 2014). Most cut flowers are produced in developing countries, whereas their sales are mainly concentrated in developed countries. Therefore, long-distance transportation from the production region to the point of sale is the mainstream channel for cut rose trade. However, long-distance transportation, usually in darkness under, low temperature, and without water, can easily trigger a massive burst of exogenous ethylene production (Faragher et al., 1987; Mor et al., 1989; Reid et al., 1989; Noodén and Schneider, 2004). Furthermore, stress-induced exogenous ethylene accelerates flower opening and senescence and decreases the vase life of cut rose by coupling with the mechanical damage during postharvest treatment (Müller et al., 2000). Therefore, avoiding and reducing the negative effects of long-distance transportation is crucial in maintaining the postharvest quality of cut roses.

As sessile organisms, plants have developed intricate mechanisms to defend themselves against external adversities, including wounding and hormones, and other biotic, and abiotic stresses. Plants are also able to sense injured tissues and protect themselves by regulating defense system-related genes. The endogenous signals released from wounded tissues may act to activate the plant defense system, and wounding-induced responses are distributed in both the site of damage (local response) and systemically (systemic response), mediated by hormone signaling, including jasmonic acid (JA), ethylene (Eth), brassinolide (BRs), salicylic acid (SA), and abscisic acid (ABA).

Previous studies revealed that mechanical damage results in a large increase in the content (~25-fold) of endogenous JA and JA-isoleucine (JA-Ile) within 5 min of tissue injury (Chung et al., 2008; Glauser et al., 2008; Koo et al., 2009). The phytohormone, JA, is a well-characterized signaling molecule for its role in protection against wounding damage (Leon et al., 2001); it also plays a central role in activating the systemic defense in many vital processes, including flower development and senescence (Wasternack, 2007). The transcript levels of several JA biosynthesis-related genes, including *LIPOXYGENASE 2* (*LOX2*), *LIPOXYGENASE 3* (*LOX3*), and *ALLENE OXIDE SYNTHASE* (*AOS*), are upregulated together with the JA acceleration (Bell et al., 1995). Arabidopsis genes such as *LOX2, AOS*, and *VEGETATIVE STORAGE PROTEIN* (*VSP*) were found to be induced by IAA (Indoleacetic Acid), and endogenous auxin content also showed a decline after wounding, showing a significant cross-talk between the two hormone-dependent signaling pathways (Devoto and Turner, 2003). The *rea1* (RAP2.6 expresser in shoot apex) mutants show a constitutive cell death phenotype, and early wounding response requires an intact JA signaling pathway (Zou et al., 2016); however, the application of methyl jasmonate (MeJA) has been shown to provide significant protection against cell death (Overmyer et al., 2000).

Ethylene is another essential systemic signal of protection of plants against wounding damage. Ethylene released from wounded plant tissues and its participation in wounding response signaling has been extensively studied for decades. Upon wounding at harvest, ethylene was rapidly synthesized in the damaged stem tissue as a result of the marked increase in 1-aminocyclopropane-1-carboxylic acid (ACC) synthase activity and the increased abundance of its transcripts. In melons, the ACC oxidase gene (*CmACO1*), the encoding enzyme controlling the last step of ethylene biosynthesis in plants, was rapidly induced (within 10 min) by ethylene treatment or upon wounding in leaves. 1-methylcyclopropene (1-MCP), a powerful inhibitor of ethylene action, significantly inhibited the accumulation of ethylene-induced *CmACO1* messenger RNA (mRNA) transcripts, while the findings did not apply to wounding-induced *CmACO1* expression (Bouquin et al., 1997). Previous pieces of evidence indicated that the transcripts of several genes encoding ACC oxidase (ACO) and ACC synthase (ACS) were rapidly and transiently accumulated after mechanical injury (Kato et al., 2002). In some cases, the enrichment of *ACS* transcripts was detected within a few hours, even as quickly as 10 min after touching or mechanical wounding in mung bean or soybean plants, respectively (Liu et al., 1993; Botella et al., 1995). Similarly, 1-aminocyclopropane-1-carboxylic acid oxidase (ACO*)* mRNA levels were also induced within several hours, while some were generally induced at a shorter period (20 or 30 min) (Balague et al., 1993; Kim and Yang, 1994; Jones and Woodson, 1999).

An increasing number of studies have shown that many members of the WRKY family are involved in multiple plant processes, such as stress defense responses, plant senescence, wounding, seed dormancy, and seed germination (Rushton et al., 2010; Birkenbihl et al., 2012; Rinerson et al., 2015; Zhao M. et al., 2020). Moreover, functional studies have shown that WRKY transcription factors (TFs) are involved in regulating senescence by interacting with JA, ABA, auxin, gibberellins (GAs), and SA-mediated signaling. In wheat, TaWRKY40-D positively regulated leaf senescence following JA and ABA treatment (Zhao L. et al., 2020). Another WRKY TF, TaWRKY42-B, interacted with and promoted the JA biosynthesis gene (*AtLOX3*, and its ortholog-*TaLOX3*), resulting in an increased JA content and an initiation of leaf senescence in Arabidopsis and wheat (Zhao M. et al., 2020). In Arabidopsis, *AtWRKY54* and *AtWRKY70* functioned redundantly in regulating leaf senescence, as revealed by single and double mutant studies (Besseau et al., 2012). Concerning the diverse roles of WRKY TFs, *AtWRKY57* functioned as a repressor in the JA-induced senescence, which was downregulated by JA and upregulated by auxin (Jiang et al., 2014). *AtWRKY45* played a positive role in the regulation of leaf senescence via GA-mediated signaling (Chen et al., 2017). Genetic analysis revealed that the crosstalk of *AtWRKY75*- SA/ROS functions correlatively in age-dependent leaf senescence (Guo et al., 2017). Finally, eight SlER-WRKYs-SlWRKY16, 17, 22, 25, 31, 33, 53, and 54 were reported to be responsive to ethylene during tomato fruit ripening (Wang et al., 2017). Taken together, WRKY proteins play critical roles in regulating plant senescence.

Ethylene is required for the signal transduction pathway in injury. However, the components involved in the initial wounding signal perception and transmission leading to ethylene synthesis are not clear. In addition, the detailed molecular mechanisms of the action between ethylene and wounding to regulate the onset of floral senescence remain poorly understood. In relation to this, identifying the initial signal of wounding and signal crosstalk between wounding and the ethylene pathway are crucial steps in understanding the early onset of floral senescence. Here, we monitored the status of the flower opening and senescence after the wounding, ethylene gas, or dual treatment daily. Furthermore, we employed RNA-sequencing (RNA-seq) and weighted gene co-expression network analysis (WGCNA) assays to investigate the signal crosstalk involvement of wounding and the ethylene pathway in the senescence process of rose flowers, and screened a WRKY transcription factor, RhWRKY33, which closely responds to wounding- and ethylene signaling. A biological analysis revealed that *RhWRKY33* plays a positive role in regulating petal senescence. In summary, our findings highlight that *RhWRKY33* mediates petal senescence by integrating wounding and ethylene signaling (a leading role).

## Materials and Methods

### Plant Materials

Cut roses (*Rosa hybrida* cv. Samantha) were grown in a greenhouse at the China Agricultural University (Beijing, China) and harvested at the second stage of a flower opening. The pretreatment methods and user definitions of flower opening stages were performed based on a previous description (Ma et al., 2006).

For the ethylene treatment, the cut roses and petal discs were exposed to gaseous ethylene (10 ppm) in an airtight growth container for 24 h (Ma et al., 2006). An aqueous solution of sodium hydroxide (NaOH) (1 M) was also placed into the sealed container to absorb the accumulated carbon dioxide (CO_2_) as previously described (Ma et al., 2005; Wu et al., 2017). For the wounding treatment, the outermost layer petals of the cut rose were wounded with a dissecting needle across its surface, and the petal discs were wounded and slightly squeezed. Then, the gas-treated and wounded cut rose or discs were incubated under the following conditions: 22 ± 1°C, 16 h/8 h photoperiod, and ~60% relative humidity. Three whole outermost layer petals of the cut rose and petal discs were harvested for RNA extraction.

### Total RNA Extraction and RNA-Seq Library Preparation

Total RNA was extracted from the rose petals as previously described (Tian et al., 2014; Wu et al., 2017), and removed from genomic DNA contamination using RNase-free DNase I (Promega); the quality and quantity were evaluated using a NanoPhotometer® spectrophotometer (Implen, California, USA) and an Agilent 2100 Bioanalyzer (Agilent Technologies, California, USA). The RNA integrity number (RIN) of all the samples (>8.0) was used for RNA-seq. The RNA-seq was performed by Beijing Novogene Bioinformatics Technology Co., Ltd. (Beijing, China). Three micrograms of the total RNA per sample were used as the input material for cDNA library construction and Illumina sequencing, and the RNA-seq data were processed, assembled, and annotated as previously described (Yang et al., 2015). Briefly, the clean RNA-seq reads were aligned to the reference genome (*Rosa chinensis* cv. Old Blush, GenBank ID 8255808) and analysis was performed using HISAT2 (version 2.0.4) (Acosta et al., 2005) for the reads derived from mRNA fragments using default parameters. The mapped reads of each sample were assembled using Stringtie (v2.1.5) (Pertea et al., 2015), and functional annotations were accomplished by searching against the National Center for Biotechnology Information (NCBI) non-redundant protein (Nr) database using the Basic Local Alignment Search Tool (BLAST)x with an E-value threshold of ≤ 10^−5^.

### Differential Expression Analysis

Differentially expressed genes between mock and other treatment conditions were performed using the DESeq R package (version 1.10.1) (Anders and Huber, 2010). The DESeq provides statistical routines for determining differential expressions in digital gene expression data using a model based on a negative binomial distribution. The resulting *p* values were adjusted using Benjamini and Hochberg's approach to control the false discovery rate. The differentially expressed genes (DEGs) were identified by DESeq, with fold changes ≥ 2 and adjusted *p-*value ≤ 0.05. All raw data were deposited in the NCBI BioProject database under the accession number PRJNA550484 (https://www.ncbi.nlm.nih.gov/bioproject/?term=PRJNA550484).

### Gene Ontology (GO) and Kyoto Encyclopedia of Genes and Genomes (KEGG) Enrichment Analysis of DEGs

The Blast2GOsoftware package was used to identify the GO enriched terms (Conesa et al., 2005). The GO terms with a corrected FDR < 0.05 were considered significantly enriched in DEGs (Conesa et al., 2005). The KEGG Orthology-Based Annotation System (KOBAS) software was used to test the statistical pathway enrichment of DEGs among the KEGG pathways (http://www.genome.jp/kegg/) (Mao et al., 2005).

### MapMan Visualization

To identify the gene functions that may play essential roles in regulating the onset of floral senescence, a MapMan visualization was performed as described previously (Thimm et al., 2004). Based on the Mercator with a blast cutoff of 50, contigs were classified into a set of hierarchical functional categories (BINs) (Lohse et al., 2014). Facing classification problems with one unigene that might have multiple contigs, the functional term of unigene was determined by the highest bit score (Nham et al., 2015). Enrichment analysis was performed using Fisher's test and Mefisto (http://www.usadellab.org/cms/index.php?page=mefisto) with Bonferroni correction. Gene expression changes were assessed using MapMan 3.5.1 R2.

### Ethylene Production Measurements

The petals of each flower were collected and placed in an airtight container (13 ml). The containers were capped and incubated for 1 h at 25°C. Then, a gas sample of 1 ml was withdrawn, using a gas-tight hypodermic syringe, and injected into a gas chromatograph (GC 17A, Shimadzu, Kyoto, Japan) for the ethylene concentration measurement (Ma et al., 2006). All measurements were performed with three replicates.

### Co-expression Analysis of Protein-Coding Genes With RNA-Seq Data

The WGCNA R software package (Langfelder and Horvath, 2008) was used to test whether the expression of protein-coding genes correlated with the senescence degree of rose flowers under the different treatments.

### Virus-Induced Gene Silencing (VIGS)

The virus-induced gene silencing of *RhWRKY33* in rose petal discs was performed as previously described (Zhang et al., 2019a). A specific fragment of *RhWRKY33* (417 bp in length) was cloned and inserted into the pTRV2 vector using X*baI* and K*pnI* sites. The recombined-TRV2 vector, pTRV2, and pTRV1 were transformed into *Agrobacterium tumefaciens* strain GV3101, and the transformed *A. tumefaciens* cells were cultured in an LB medium (supplemented with 50 μg/ml kanamycin and 50 μg/ml rifampicin) on a rocking platform (200 rpm) at 28°C for 18 h. Cells were harvested and resuspended in an infiltration buffer (10mM MgCl_2_, 200 mM acetosyringone, 10 mM MES, pH 5.6) to a final OD 600 of ~1.0. The culture mixtures contained pTRV1- and pTRV2-*RhWRKY33* at a ratio of 1:1 (v/v) or pTRV1 and pTRV2 (negative control). The culture mixtures were incubated in the dark at room temperature for 3–4 h before infiltration. Discs (1 cm diameter) were submerged in the infiltration buffer and were exposed to a vacuum (−25 kPa) two times (each for 5 min), washed with deionized water, and then incubated in the dark at 8°C for 3 days. The phenotypes of the discs were observed and counted.

### RT-qPCR

The genomic DNA of the total RNA samples was removed using RNase-free DNase I (Promega). Reverse transcription was performed with 1 μg of the total RNA using HiScript II Q Select RT SuperMix for qPCR (+gDNA wiper) (Vazyme, Nanjing, China). Together with 2 μl [20ng] cDNA used as a template, RT-qPCR was performed using a StepOne Real-Time PCR System (Applied Biosystems, California, USA) with a Kapa SYBR Fast Universal qPCR Kit (Kapa Biosystems, Boston, Massachusetts, USA). Rose *RhUBI2* and *RhActin5* were used as reference genes (Bustin et al., 2009; Meng et al., 2013; Pei et al., 2013; Gao et al., 2016; Wu et al., 2017; Zhang et al., 2019b, 2021), and the mean of reference genes was used in the following analysis to avoid discrepancies; Relative quantification of the transcript abundance of each gene was performed using the 2^−ΔΔCt^ method (Livak and Schmittgen, 2001). All primers are listed in Supplementary Table 6.

### Statistical Analyses

GraphPad Prism 8.0.2 software (http://www.graphpad.com/) was used to analyze the statistical differences in the data. Two groups of data were compared by using two-sided Student's *t*-tests (^*^p < 0.05, ^**^p < *0.0*1, ^***^p < 0.001, ^****^p < 0.0001). The means of the multiple groups of data were compared *via* one-way ANOVA and Bonferroni's *post-hoc* test, with *p* < 0.05 considered significant.

## Results

### Wounding Accelerates Rose Floral Senescence Dependent on Ethylene Signaling

**Figure 1:**
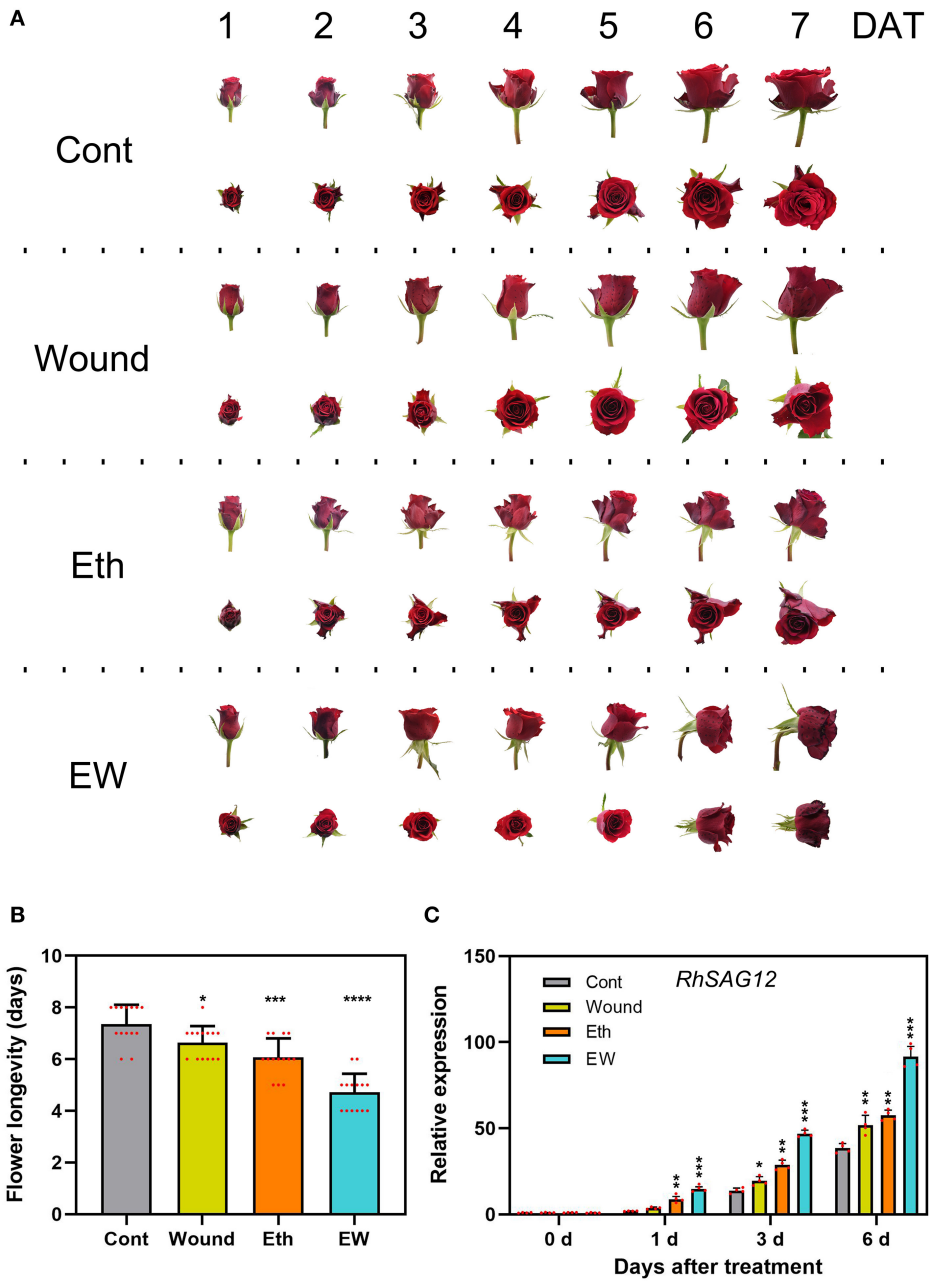
Effects of wound and ethylene on the senescence of rose flowers. **(A)** The phenotypes of flowers were recorded and photographed every day after wound and ethylene treatment. **(B,C)** The flower longevity and relative expression of *RhSAG12* by reverse transcription PCR (RT-qPCR) were determined. Values are means ± SD, *RhUBI2* and *RhActin5* were used as reference genes, and asterisks indicate statistically significant differences (*p < 0.05, **p < 0.01, ***p < 0.001, Student's *t*-test).

The role of ethylene in the acceleration of floral senescence has been reported (Ma et al., 2018), but limited research has been conducted on the function of wounding in petal senescence. To determine the effects and signaling crosstalk between wounding and ethylene during floral senescence, we studied the lifespan of cut rose flowers under different treatments. Results showed that the wounded cut roses displayed a slight wilting until 7 days after treatment (DATs); their lifespan was 6.6 ± 0.6 days and there existed significant differences compared with the controls (7.4 ± 0.7 days) (Figures 1A,B). The rose flower that underwent ethylene treatment showed severe wilting after 6 days, and after the dual treatment with wounding and ethylene, the wilting started at 4 DAT, and nearly all flowers displayed complete wilting after 6 DAT. The life cycle of the cut rose that underwent the dual-treatment by wounding and ethylene was 4.7 ± 0.7 days, which showed more severe wilting than those that dealt with single treatments (Figures 1A,B). Furthermore, the expression level of senescence-associated gene *RhSAG12*, a molecular marker of rose floral senescence progression, was significantly higher in the dual-treated flowers compared with the control-treated flowers at 1, 3, and 6 DAT (Figure 1C). Moreover, we measured the ethylene production under different treatments, and we found that the ethylene production in the wound-treated petals (4.2 ± 0.7 C_2_H_4_ g^−1^ FW h^−1^) was higher than that in the control (2.2 ± 0.6 C_2_H_4_ g^−1^ FW h^−1^) but lower than that in the ethylene (4.5 ± 0.8 C_2_H_4_ g^−1^ FW h^−1^) or wounding- and ethylene- (EW) dual treatment (6.9 ± 0.9 C_2_H_4_ g^−1^ FW h^−1^) at 6 DAT (Supplementary Figure 1). Briefly, these results indicate that the single wounding or ethylene treatment could significantly accelerate the senescence process of rose flowers to some content; however, the role of the wounding was of a senescence-facilitating factor in ethylene signaling.

### Global Analysis of Transcriptome Data

To determine the alteration in gene expression during the onset of flower senescence under the dual treatment with wounding and ethylene, we extracted RNA samples from the four different treatments with three biological replicates. The RNA sequencing produced 48,033,008; 55,887,823; 57,773,772; and 58,588,791 clean reads from the four different treatments, respectively. Sequences were mapped to the reference genome (*Rosa chinensis* cv. Old Blush, GenBank ID 8255808) for annotation, and the mapping rate reached over 83% for the samples from each treatment (Supplementary Table 1). Differentially expressed genes were screened by comparing three different treatments with the control treatment, and 6,771 unigenes that were differentially expressed across these treatments were generated (fold changes ≥ 2, *p* ≤ 0.05). The number of DEGs was counted as 274 from Group 1 (wounding vs. control), 2,442 from group 2 (ethylene vs. control), and 4,055 from group 3 (EW vs. control) (Supplementary Figure 2A). Results showed that the three groups that shared 96 DEGs attributed to the wounding, drawing few gene expression changes, which was consistent with the single wounding not accelerating the floral senescence process. In addition, Groups 2 and 3 shared 2,093 DEGs, and Group 3 specifically induced 1,903 DEGs (Supplementary Figure 2B), both of which had enriched functional categories related to protein degradation, hormone metabolism, and transcription regulation.

### Complex Hormone Biosynthesis and Signaling Pathways Involved in Wounding and Ethylene Signaling

**Figure 2:**
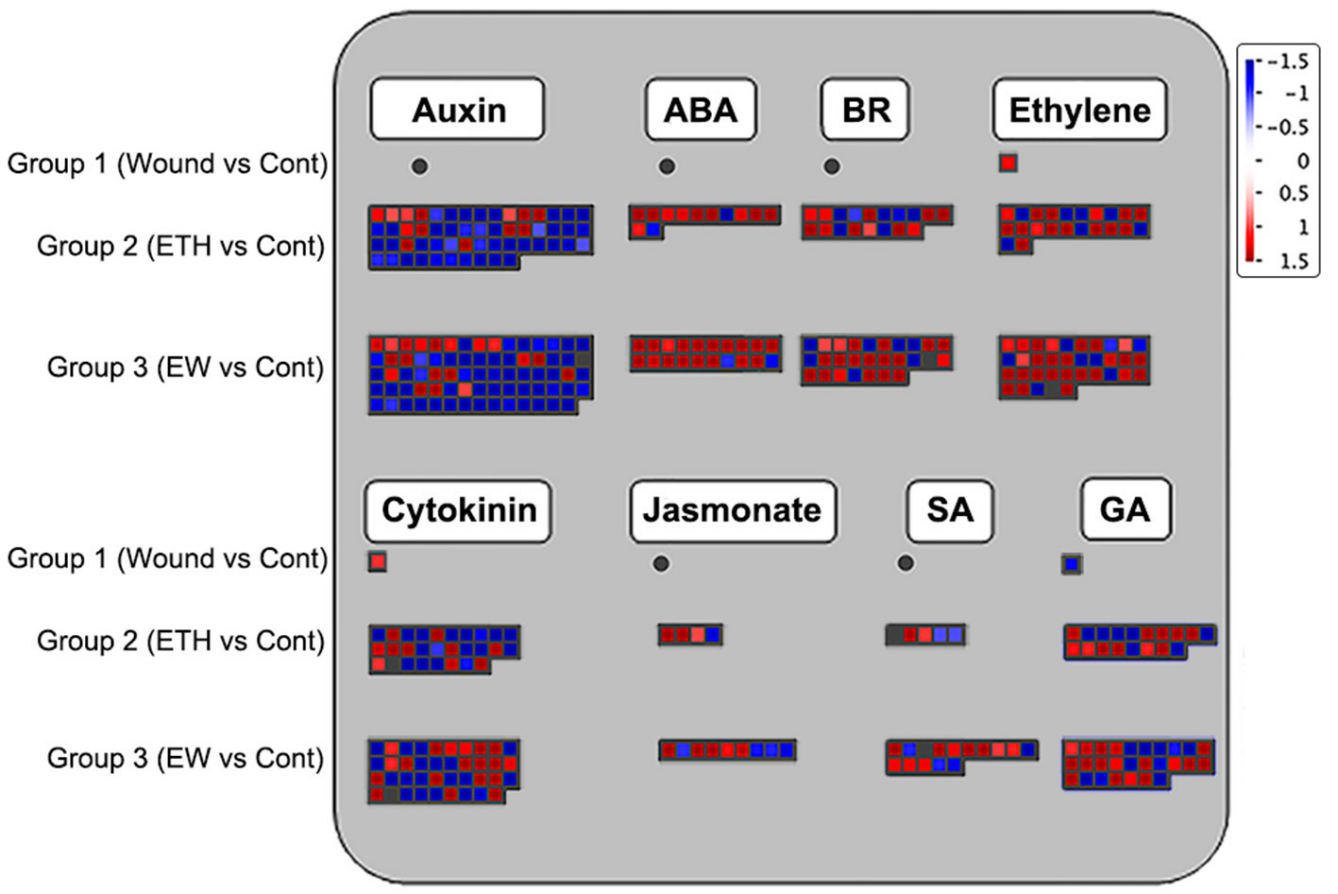
Display of differentially expressed genes (DEGs) involved in hormone biosynthesis and signaling transduction pathway. Significant DEGs (fold changes ≥ 2 and adjusted *p*-value ≤ 0.05) were organized into functional categories (BINs) (Thimm et al., 2004) and visualized using the MapMan software (version 1.0). The color set scale was placed on the top right, and the gene expression increased is indicated in red and decreased in blue. Detailed information on gene expression is listed in Supplementary Table 2.

To further understand the detailed regulatory network during the onset of rose floral senescence under wounding and ethylene treatments, all DEGs related to hormone biosynthesis and signaling pathways across the three groups were analyzed using the MapMan software. The largest numbers of DEGs were related to biosynthesis and signal transduction of auxin and ethylene, followed by DEGs in the cytokinin pathway (Figure 2; Supplementary Table 2).

For the auxin pathway, the vast majority of DEGs were downregulated after the wounding and ethylene treatments. For example, 76.4% of the DEGs (42/55) were downregulated in Group 2 and 74.3% (55/74) in Group 3. Among these genes, 52 DEGs were co-regulated by the ethylene and wounding- and ethylene- dual treatments, including 35 small auxin upregulated RNA (SAUR) and SAUR-like genes, two PIN-FORMED (PIN) family genes (rchiobhmchr2g0169101 and rchiobhmchr3g0448071), and four auxin-responsive GH3 family proteins (rchiobhmchr2g0107081, rchiobhmchr2g0107091, rchiobhmchr2g0162261, and rchiobhmchr4g0428671). There were 22 DEGs that responded specifically to EW treatment related to auxin biosynthesis and signal transduction. Most DEGs related to the ethylene pathway were upregulated in Groups 2 and 3, with the proportions of 68.2% (15/22) and 77.1% (27/35), respectively. In the ethylene biosynthesis pathway, including ACC oxidase (rchiobhmchr5g0001831 and rchiobhmchr3g0474401), two ethylene receptors (rchiobhmchr4g0391281 and rchiobhmchr6g0294491), and three ERF genes were upregulated, whereas eight ERF genes were downregulated in Groups 2. Among the genes related to the cytokinin signaling pathway, ~39.3% (11/28) and 51.3% (20/39) of DEGs were increased in groups 2 and 3, respectively. Cytokinin synthase IPT3/5 genes (rchiobhmchr5g0029421 and rchiobhmchr4g0418401) and cytokinin oxidase CKX6/7 genes (rchiobhmchr1g0319331 and rchiobhmchr6g0301781) were identified and upregulated in Groups 2 and 3, whereas the expression of cytokinin oxidase CKX3 was specifically enhanced in Group 3.

Differentially expressed genes related to ABA, BRs, JA, SA, and GA were also identified. For the ABA pathway, a total of 11 genes, including the biosynthesis genes AAO3 (rchiobhmchr5g0049511), NCED (rchiobhmchr4g0397001 and rchiobhmchr5g0027901), and signal transduction genes PP2C (rchiobhmchr2g0094051), were regulated by the ethylene or EW treatments. A total of 27 DEGs were found in Group 3, referred to as brassinosteroid biosynthetic and signaling pathways, these included biosynthetic genes DEF4 (rchiobhmchr2g0101791), CYP90D1 (rchiobhmchr2g0142901), CAS1 (novel00019), and signaling transduction genes BEH4 (rchiobhmchr5g0011971) and BRH1 (rchiobhmchr7g0197001). Among the JA-related genes, the upregulated DEGs, including AOS2 (rchiobhmchr6g0261031, rchiobhmchr6g0261011, and rchiobhmchr6g0300221), OPR2 (rchiobhmchr2g0167371), and LOX1 (rchiobhmchr3g0455111), were related to biosynthesis and detected in Group 3. In the SA pathway, the expression of UDP-glucosyltransferase gene UGT74E2 (rchiobhmchr6g0248381, rchiobhmchr6g0248411, rchiobhmchr7g0179611, and rchiobhmchr6g0248381) was enhanced, whereas SABATH methyltransferase expression (novel01210 and rchiobhmchr6g0265391) was decreased in Group 3. The expression of eight GA biosynthetic and four signal transduction genes showed changes in Groups 2 and 3. Among those DEGs, the biosynthetic genes were GA2 (rchiobhmchr5g0023471), GA20ox1 (rchiobhmchr1g0351591 and rchiobhmchr1g0376161), GA3ox1/2 (rchiobhmchr7g0236321 and rchiobhmchr2g0137231), GA2ox1/2/6 (rchiobhmchr5g0027981, rchiobhmchr5g0014211, andrchiobhmchr4g0426161), and signaling pathway genes, including GAI (rchiobhmchr2g0089571), GID1B (rchiobhmchr6g0292241), and GASA6 (rchiobhmchr7g0181391). Briefly, we speculated that different hormone biosynthesis and signaling pathways may be involved in the senescence process induced by wounding and ethylene.

### Differential Gene Expression Related to Transcription Factors, Protein Modification, and Degradation

**Figure 3:**
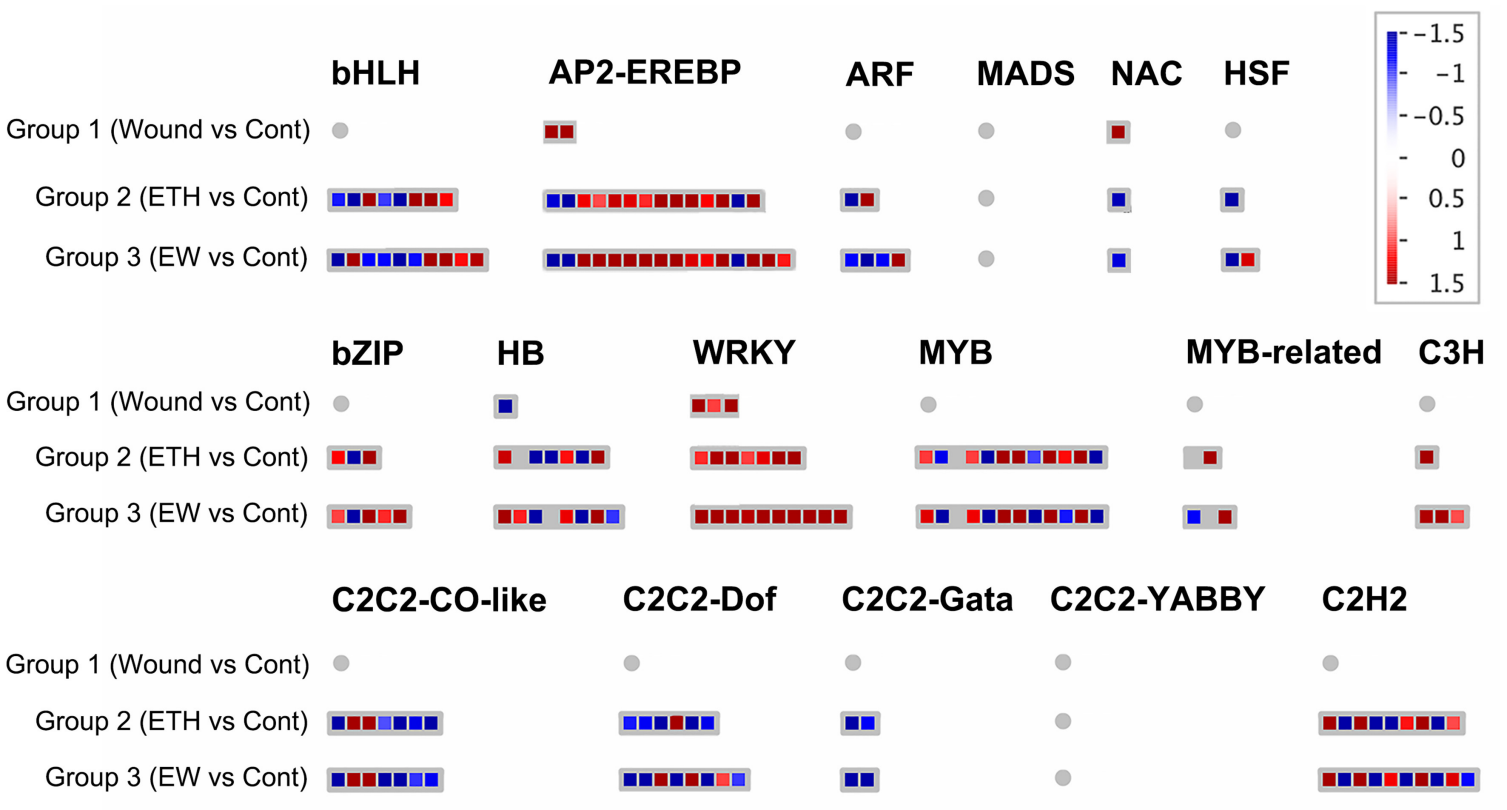
Display of differentially expressed genes related to transcription factors. Significant DEGs (fold changes ≥ 2 and adjusted *p*-value ≤ 0.05) were organized into functional categories (BINs) (Thimm et al., 2004) and visualized by MapMan software (version 1.0). The color set scale was placed on the top right, and the gene expression increased is indicated in red and decreased in blue. Detailed information on gene expression is listed in Supplementary Table 3.

In terms of transcription factors, a total of 1,123, and 222 DEGs encoding TFs were observed in Groups 1, 2, and 3, respectively (Figure 3; Supplementary Table 3). Major differentially expressed TF families included WRKY, MYB, bHLH, HB, bZIP, AP2-EREBP, and C2H2. The predominant family was AP2-EREBP TF, with 14/16 DEGs, including 11/13 upregulated genes in Groups 2 and 3, respectively, followed by MYB (12/12), C2H2 type-zinc finger protein (9/10), bHLH (8/10), WRKY (7/10), HB (7/8), and bZIP (3/5) families. These data suggest that the TFs above may be involved in EW signaling during flower senescence.

### Screening of Candidate Genes Based on Co-expression Network

**Figure 4:**
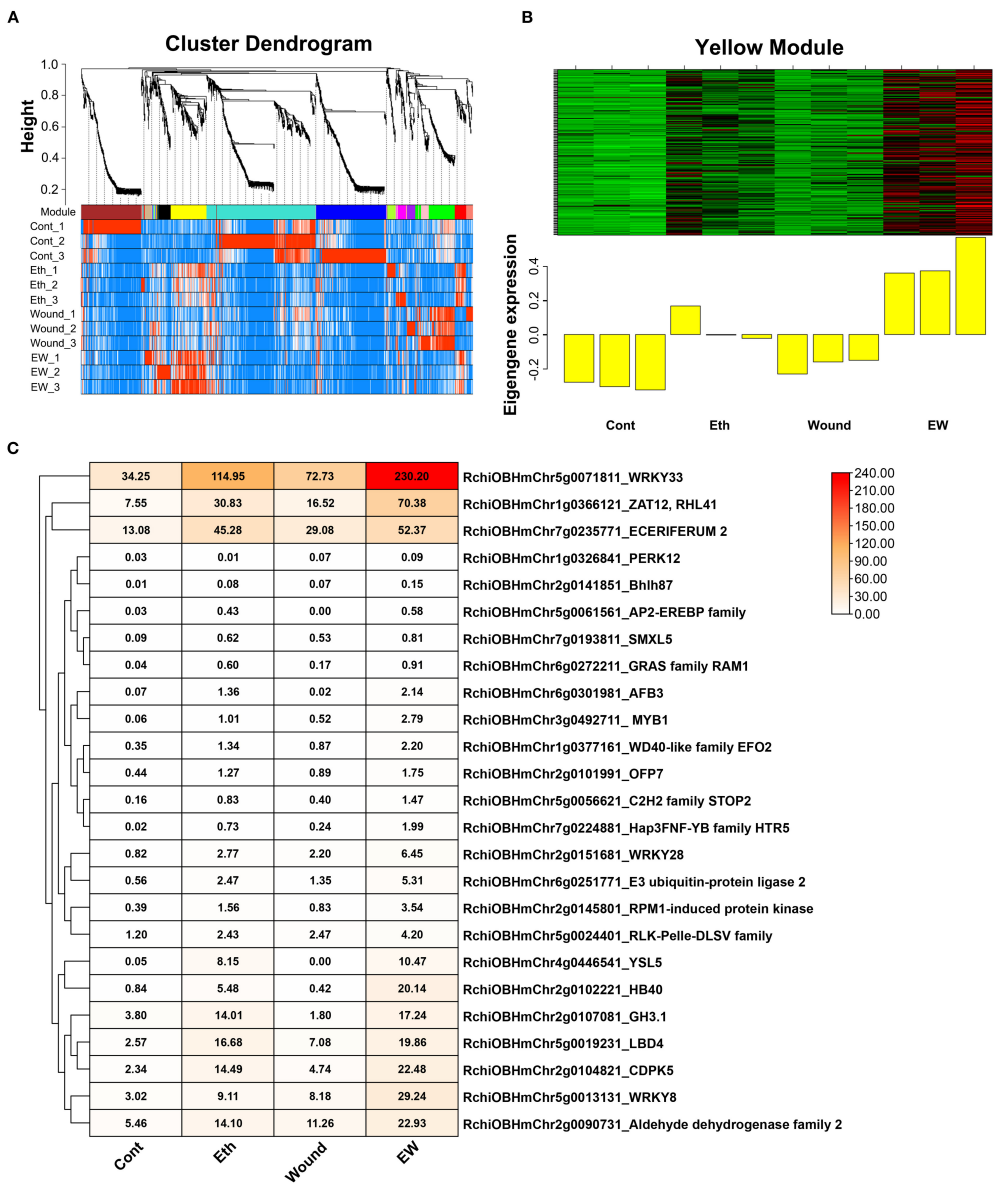
Co-expression network analysis of DEGs involved in wound and ethylene signaling. **(A)** Clustering dendrogram of genes across all samples, with dissimilarity based on the topological overlap, together with assigned merged colors and original module colors. **(B)** Heatmap of the eigengene expression in the “yellow” module in response to wound and ethylene signaling. **(C)** Expression profile of screened hub genes in different treatments.

To further identify the major groups, we performed a WGCNA and obtained 13 distinct modules (Figure 4A). Focused on the rose senescence degree, we found that the “Yellow” module was the most significantly associated with rose senescence (Figure 4B). Moreover, we analyzed the expression profile of hub genes in the “Yellow” module and found that *RchiOBHmChr5g0071811* was the most significantly upregulated gene when compared with control (Figure 4C).

Phylogenetic and protein sequence analyses were performed (Supplementary Figures 3, 4), and RchiOBHmChr5g0071811 was closely related to AtWRKY33, which contains two WRKY domains. Furthermore, it was located in the nucleus by subcellular localization in *Nicotiana benthamiana* (Supplementary Figure 4B). Based on these results, *RchiOBHmChr5g0071811* was named *RhWRKY33*. RhWRKY33 accumulates in the nucleus, where it may function as a transcription factor.

To better understand its expression characteristics, the transcription level of *RhWRKY33* was measured in different organs and petal stages through RT-qPCR. As shown in Supplementary Figure 4C, the expression level of *RhWRKY33* was higher in the sepals, petals, and stamens, and lower in young leaves, receptacles, and pistils, whereas it significantly increased from stage 1 to stage 5 (Supplementary Figure 4D), implying that *RhWRKY33* may function in rose petal senescence.

### *RhWRKY33* Accelerates the Petal Senescence Depended on Wounding and Ethylene Signaling

**Figure 5:**
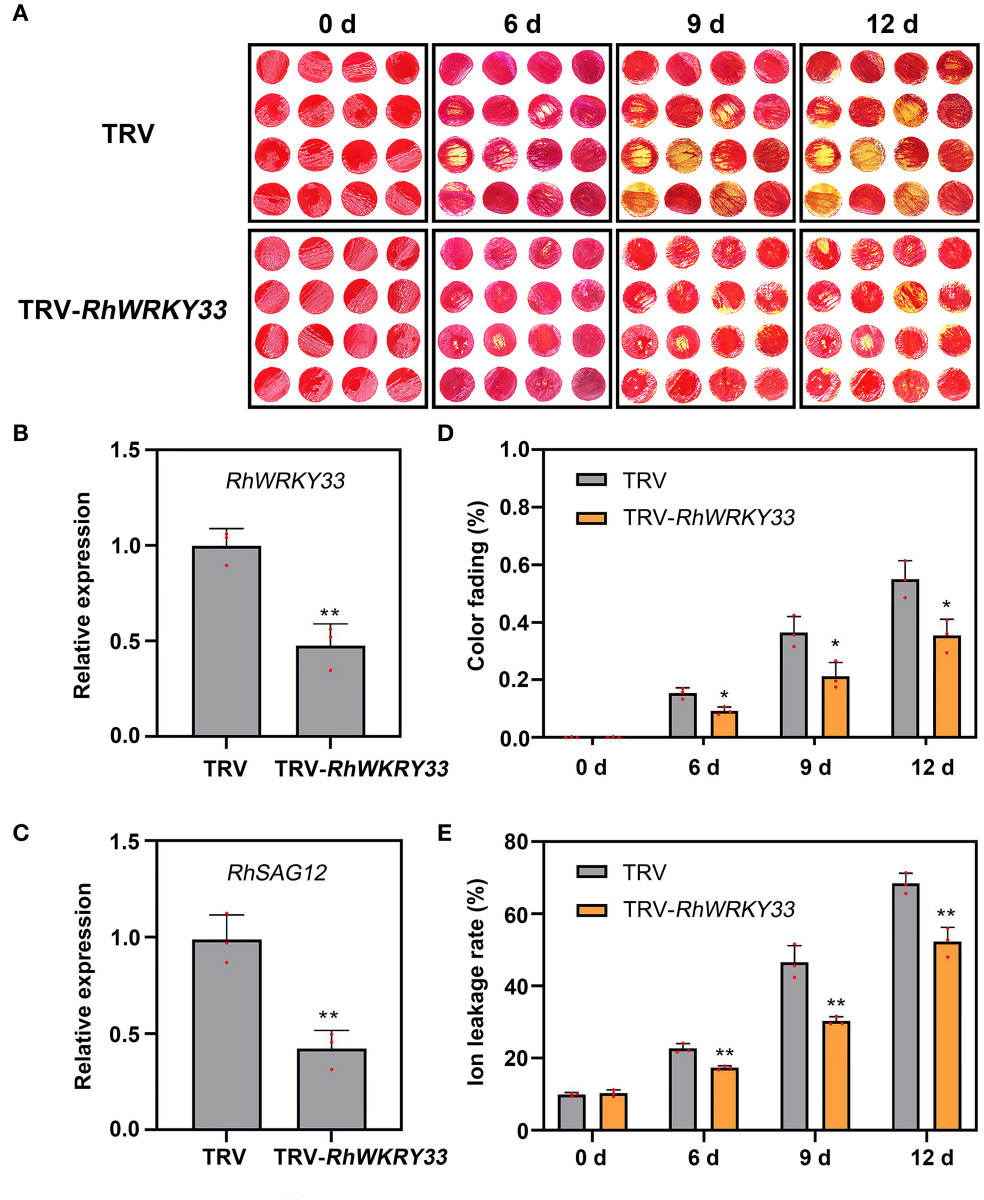
Effect of TRV-*RhWRKY33* on the senescence of rose petal discs. **(A)** Color fading phenotypes of TRV and TRV-*RhWRKY33* petal discs. **(B)** RT-qPCR validation of expression level of *RhWRKY33* in TRV and TRV-*RhWRKY33* petal discs. **(C)** *RhSAG12* expression levels were analyzed in *RhWRKY33*-silenced and TRV control petal discs. **(D)** Color fading rate. **(E)** Ion leakage rate. The error bars represent the means of three biological replicates ± SDs, *RhUBI2*, and *RhActin5* were used as reference genes, and the asterisks indicate significant differences based on Student's *t*-test (*p < 0.05; **p < 0.01).

**Figure 6:**
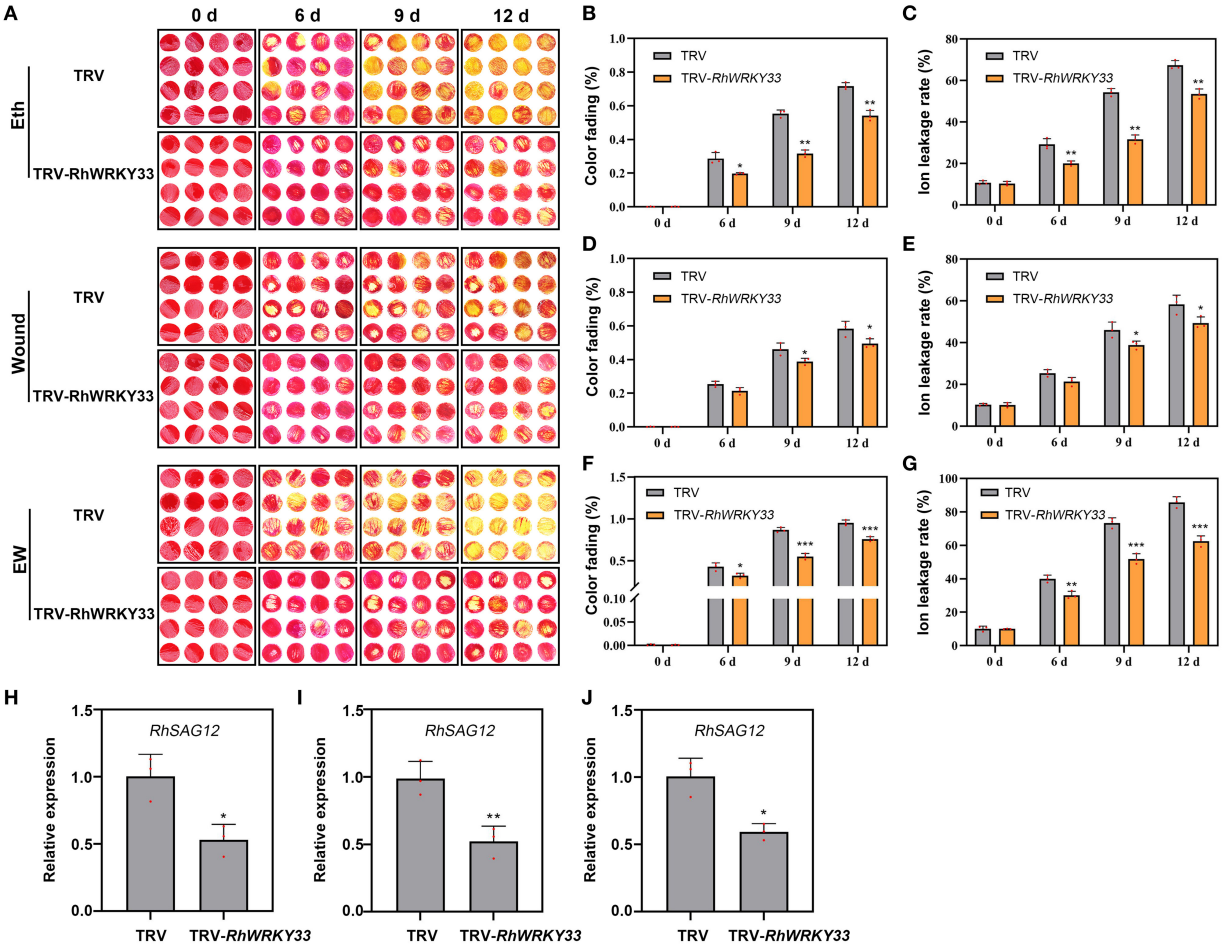
Effect of wounding-, ethylene- and EW-treatment on the senescence of *RhWRKY33*-silenced rose petal discs. **(A)** The phenotypes of rose petal discs treated with 10 μl/L ethylene, wound, or EW. **(B,D,F)** Color fading rate. **(C,E,G)** Ion leakage rate. **(H,I,J)** Expression levels of *RhSAG12* were analyzed in *RhWRKY33*-silenced and TRV control petal discs under different treatments. The error bars represent the means of three biological replicates ± SDs, *RhUBI2*, and *RhActin5* were used as reference genes, and the asterisks indicate significant differences based on Student's *t*-test (*p < 0.05; **p < 0.01; ***p < 0.001).

To evaluate whether *RhWRKY33* was involved in regulating the senescence of rose petals, we silenced the *RhWRKY33* transcripts in petal discs by VIGS and found that the color fading of TRV discs began after 6 days, whereas the TRV-*WRKY33* discs, with *WRKY33* silenced, did not display any significant color fading (Figure 5A); in contrast, the degree of the TRV disc fading was more serious after 9 and 12 days compared with the TRV-*WRKY33* discs (Figure 5D). The expression of *RhWRKY33* was significantly lower in the TRV-*WRKY33* discs than in the TRV control discs (Figure 5B); Concurrently, we measured the expression of *RhSAG12* and ion leakage in TRV and TRV-*WRKY33* discs at 0, 6, 9, 12 days. Consistent with this, the *WRKY33-*silenced discs presented lower expression of *RhSAG12* and less ion leakage than TRV control (Figures 5C,E). These data provide evidence that *RhWRKY33* plays a positive regulatory role during rose petal senescence.

further investigate whether *RhWRKY33* was involved in EW signaling, we also performed ethylene, wounding, and EW treatments through the VIGS assay. As shown in Figures 6A–G, the fading color and ion leakage were significantly increased in the ethyne and EW treatment compared with wounding-treatment at 6, 9, and 12 DAT, either in TRV or in TRV-*RhWRKY33*, suggesting the stronger role of ethylene than wounding on petal senescence. Irrespective of the wounding, ethylene, or EW treatment, all *RhWRKY33-*silenced discs exhibited longer floral longevity, lower color fading rate, and ion leakage when compared with the TRV. In addition, the expression of *RhSAG12* was significantly lower in *RhWRKY33*-silenced discs at 6 DAT than in TRV discs under the above treatments (Figure 6). These results indicate that *RhWRKY33* can accelerate the senescence process of rose petals depending on the wounding and ethylene signaling, but mainly on ethylene signaling.

We previously found that JA and ethylene could promote senescence in rose flowers (Zhang et al., 2019b), and JA levels increased significantly in plants subjected to wounding (Chung et al., 2008; Glauser et al., 2008; Koo et al., 2009); whether *RhWRKY33* was induced by JA remains unknown. To examine the relationship between JA and *RhWRKY33*, we measured the expression of *RhWRKY33* in JA- and ethylene-induced petals and found that *RhWRKY33* was upregulated by JA and ethylene treatment (Supplementary Figure 5). These results further indicate that *RhWRKY33* accelerates the petal senescence process mediated by both ethylene and wounding signaling, but relies heavily on ethylene signaling.

## Discussion

### Ethylene Is a Senescence-Promoter Hormone in Rose Petals

Phytohormones possess pivotal roles in initiating and modulating floral senescence programs and finally influence the quality and long vase life of fully open petals. Among those hormones, ethylene and ABA function as the senescence-accelerating factors, while auxin, CTKs, SA, and GA act the inhibited function in floral senescence (Lu et al., 2014; Trivellini et al., 2015; Wu et al., 2017; Shabanian et al., 2018). Particularly, ethylene has been considered as a major endogenous regulator of fruit ripening, leaf- and petal-senescence, and floral organ abscission (Li et al., 2013; Han et al., 2016; Shabanian et al., 2018). Once the senescence process of leaves and flowers was initiated, the expression of ethylene biosynthesis genes *ACS1/2* was strongly increased, accompanied by a massive burst of ethylene production (Ma et al., 2005). In this study, two ethylene biosynthesis genes, *ACS* (rchiobhmchr6g0310191) and *ACO* (rchiobhmchr3g0474401, rchiobhmchr5g0001831), had higher levels of expression in all the ethylene treatment flowers (group 2 and 3) (Figure 2; Supplementary Table 2). Meanwhile, we also found that the expression of eight *ERF* genes (rchiobhmchr4g0392451, rchiobhmchr4g0392501, rchiobhmchr5g0008991, rchiobhmchr5g0009711, rchiobhmchr5g0032721, rchiobhmchr6g0288231, rchiobhmchr6g0299771, and rchiobhmchr7g0204641) were increased, and three *ERF* genes (rchiobhmchr2g0135921, rchiobhmchr6g0298011, and rchiobhmchr7g0230931) were decreased in response to the ethylene signal, and *RhERF1* (rchiobhmchr2g0135921) and *RhERF4* (rchiobhmchr6g0298011) have been proved to function in petal abscission mediated by ethylene pathway (Gao et al., 2019).

The exogenous application of ethylene also triggers the gene expression change related to the biosynthesis and signaling pathway of other hormones. During the leaf senescence and abscission process, the massive production of ethylene could inhibit auxin synthesis and transport, enhance auxin degradation, and lower diffusible auxin levels (Burg, 1968). Here, up to 55 DEGs associated with the auxin pathway were induced and 76.4% of those genes (42/55) were downregulated by exogenous ethylene applying, including auxin polar transport PIN family genes (*PIN5*, rchiobhmchr3g0448071; *PIN8*, rchiobhmchr2g0169101), auxin-responsive SAUR, and the genes of GH3 family (Figure 2; Supplementary Table 2). We speculate that ethylene may directly or indirectly regulate the expression of auxin-related genes to affect flower senescence. Meanwhile, the exogenous application of ethylene further enhanced senescence-promoting hormone responses, such as ABA, and while declined the genes expression associated with synthesis and signaling transduction of cytokinin, which functions in delaying senescence.

### Wounding Function in Acceleration Floral Senescence Depend on Ethylene Signaling

Numerous studies have revealed that wounding is implicated in multiple signaling pathways, such as JA, auxin, and ethylene. Wounding is considered one of the early triggers of plant regeneration (Chen et al., 2016), and the content of JA and auxin accumulates within seconds to minutes after wounding, making them candidates for early wounding signal. Further analysis indicated that cell damage leads to JA accumulation, and tissue integrity loss leads to local auxin accumulation by impeding polar auxin transport (Glauser et al., 2009; Larrieu et al., 2015; Zhou et al., 2019). Endogenous JA levels acceleration in response to mechanical wounding and herbivore damage have been extensively reported (Creelman and Mullet, 1997; Rao et al., 2000). JA signal marker genes, namely, *LOX2, AOS*, and *VSP*, were found to be induced, whereas endogenous levels of auxins were also shown to decline after wounding, showing a significant cross-talk between the hormone and damage signaling pathways (Devoto and Turner, 2003; Zou et al., 2016). Previous studies confirmed that the damage signal is one of the primary factors in the induction of the senescence phenotype in detached leaves or petals, whereas ethylene is implicated as a regulator of the rate of the process (Philosophhadas et al., 1991). Additional evidence has revealed that ethylene content is generally induced by various environmental stresses, such as damage signaling, exposure to low temperatures, and water stress, in plants (Hyodo et al., 1991).

Our previous study revealed that *RhMYB108* could accelerate rose petal senescence by mediating JA and ethylene signaling (Zhang et al., 2019b; Fan et al., 2020). In this study, we found that the rate of petal senescence was not significantly accelerated or delayed in the single wounding treatment. However, the senescence rate of EW-treated petals was faster than those treated by wounding or ethylene, which suggested that wounding could strengthen ethylene signaling, which was consistent with those of a study by Hyodo (Hyodo et al., 1991). In conclusion, further research is needed to reveal the mechanisms underlying the wound-ethylene crosstalk in petal senescence. In addition, our study suggested that RhWRKY33 could accelerate the floral senescence process of cut roses by mediating EW-signaling (Figures 1, 6).

### *RhWRKY33* Mediates Ethylene and Wounding Signaling During Senescence

A complex network of transcription factors is involved in senescence. In terms of transcription factors, a total of 1,123, and 222 DEGs encoding TFs were observed in Groups 1, 2, and 3, respectively (Figure 3; Supplementary Table 3). Among the differentially expressed TFs, 119 DEGs responded to both Groups 2 and 3 (Supplementary Table 4), whereas 102 genes were expressed in response to Group 3 (Supplementary Table 5). Major differentially expressed TF families included WRKY, MYB, bHLH, HB, bZIP, AP2-EREBP, and C2H2. Here, AP2-EREBP TF was the predominant family with 14/16 DEGs, including 11/13 upregulated genes in Groups 2 and 3, respectively, followed by MYB (12/12), C2H2 type-zinc finger protein (9/10), bHLH (8/10), WRKY (7/10), HB (7/8), and bZIP (3/5) families.

Up to date, increasing studies have shown that many WRKY family members are involved in stress-defense responses, plant senescence, wounding, seed dormancy, and seed germination (Rushton et al., 2010; Rinerson et al., 2015; Zhao M. et al., 2020). In addition, a complex regulatory network consisting of WRKY TFs and plant hormone functions is involved in plant senescence. In our study, the expression of *RhWRKY33* was significantly upregulated more than 9 and 6 times, when induced by JA and ethylene, respectively. However, it was significantly repressed by 1-MCP (Supplementary Figure 5). We speculate that *RhWRKY33* may be induced by wounds by increasing the JA content. However, in this study, we discovered that the expression of *RhWRKY33* was upregulated more than two times under wounding treatment, and less than nine times under JA treatment. Whether there are other factors involved in the regulation of rose petal senescence requires further study.

Overall, using RNA-seq analysis and WGCNA, we identified the RhWRKY33, which is closely related to AtWRKY33 from *Arabidopsis thaliana* and is located in the nucleus. The silencing of *RhWRKY33* (TRV-*RhWRKY33*) significantly delayed the rose petal senescence process either on the control or on other treatments (ethylene, wounding, and EW). Ethylene plays a major role in petal senescence. From the above results, we conclude that RhWRKY33 is involved in regulating rose petal senescence by mediating wound- and ethylene-signaling.

## Data Availability Statement

The original contributions presented in the study are publicly available. This data can be found here: National Center for Biotechnology Information (NCBI) BioProject database under accession number PRJNA550484 (https://www.ncbi.nlm.nih.gov/bioproject/?term=PRJNA550484).

## Author Contributions

WJ and QZ performed the experiments and analyzed the data. YLi and YLiu designed the study. SZ, DZ, JX, HZ, and FW provided technical support and conceptual advice. WJ, QZ, SZ, and YLi wrote the manuscript. All authors read and approved the final manuscript.

## Funding

This study was supported by the General Project of Shenzhen Science and Technology and Innovation Commission (21K270360620) and the National Natural Science Foundation of China (Grant No. 31902054).

## Conflict of Interest

The authors declare that the research was conducted in the absence of any commercial or financial relationships that could be construed as a potential conflict of interest.

## Publisher's Note

All claims expressed in this article are solely those of the authors and do not necessarily represent those of their affiliated organizations, or those of the publisher, the editors and the reviewers. Any product that may be evaluated in this article, or claim that may be made by its manufacturer, is not guaranteed or endorsed by the publisher.

## Supplementary Material

Supplementary Figure 1 The ethylene production of different treatments was determined at 0, 1, 3, and 6 days. Values are means ± SD, and asterisks indicate statistically significant differences (^*^*p* < 0.05, ^**^*p* < 0.01, Student's *t*-test).

Supplementary Figure 2 Identification of differentially expressed genes among all treatments during the onset of floral senescence.

Supplementary Figure 3 Biochemical characteristics and spatiotemporal expression analysis of RhWRKY33.

Supplementary Figure 4 Display of DEGs relating to transcription factors, protein modification, protein degradation **(A)**, and hormone signaling **(B)** was specifically regulated by Group 3.

Supplementary Figure 5 Effects of different hormone treatments on the expression of *RhWRKY33*.

Supplementary Table 1 Statistics of annotation results for rose unigenes.

Supplementary Table 2 Detailed information of DEGs involved in hormone biosynthesis and signaling transduction pathway.

Supplementary Table 3 Detailed information of DEGs related to transcription factors.

Supplementary Table 4 Detailed information of DEGs relating to transcription factors, protein modification, protein degradation **(A)**, and hormone signaling **(B)** was co-regulated between Groups 2 and 3.

Supplementary Table 5 Detailed information of DEGs relating to transcription factors, protein modification, protein degradation **(A)**, and hormone signaling **(B)** was specifically regulated by group 3.

Supplementary Table 6 List of primers used.
