# Supplementary figures and images for: RhWRKY33 Positively Regulates Onset of Floral Senescence by Responding to Wounding- and Ethylene-Signaling in Rose Plants
Source: Front Plant Sci. 2021 Nov 5;12:726797. doi: 10.3389/fpls.2021.726797 (PMC8602865; doi:10.3389/fpls.2021.726797)

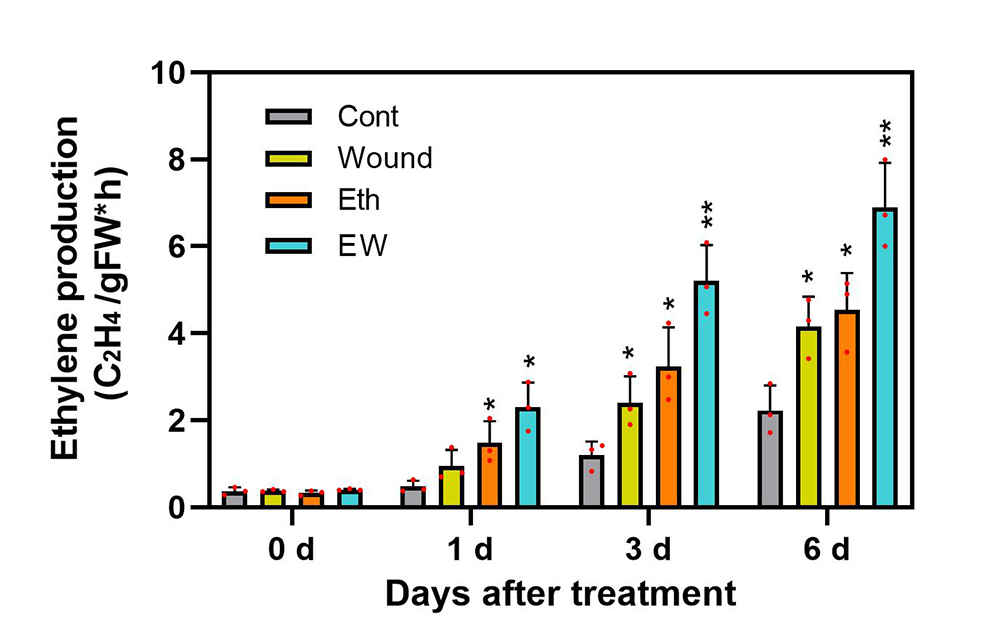

Supplement: Supplementary Figure 1 — The ethylene production of different treatments was determined at 0, 1, 3, and 6 days. Values are means ± SD, and asterisks indicate statistically significant differences (*p < 0.05, **p < 0.01, Student's t-test). [file Image_1.JPEG]

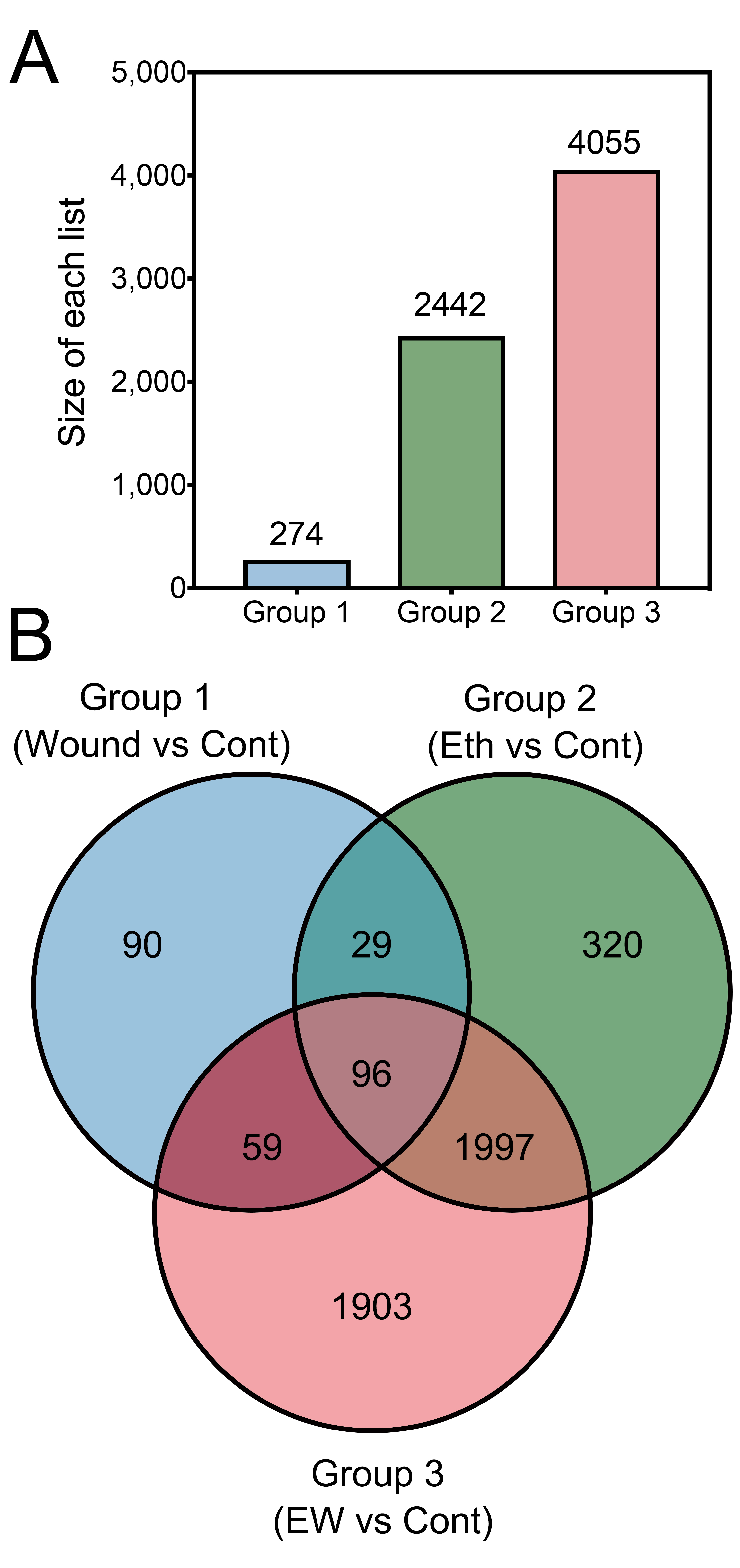

Supplement: Supplementary Figure 2 — Identification of differentially expressed genes among all treatments during the onset of floral senescence. [file Image_2.JPEG]

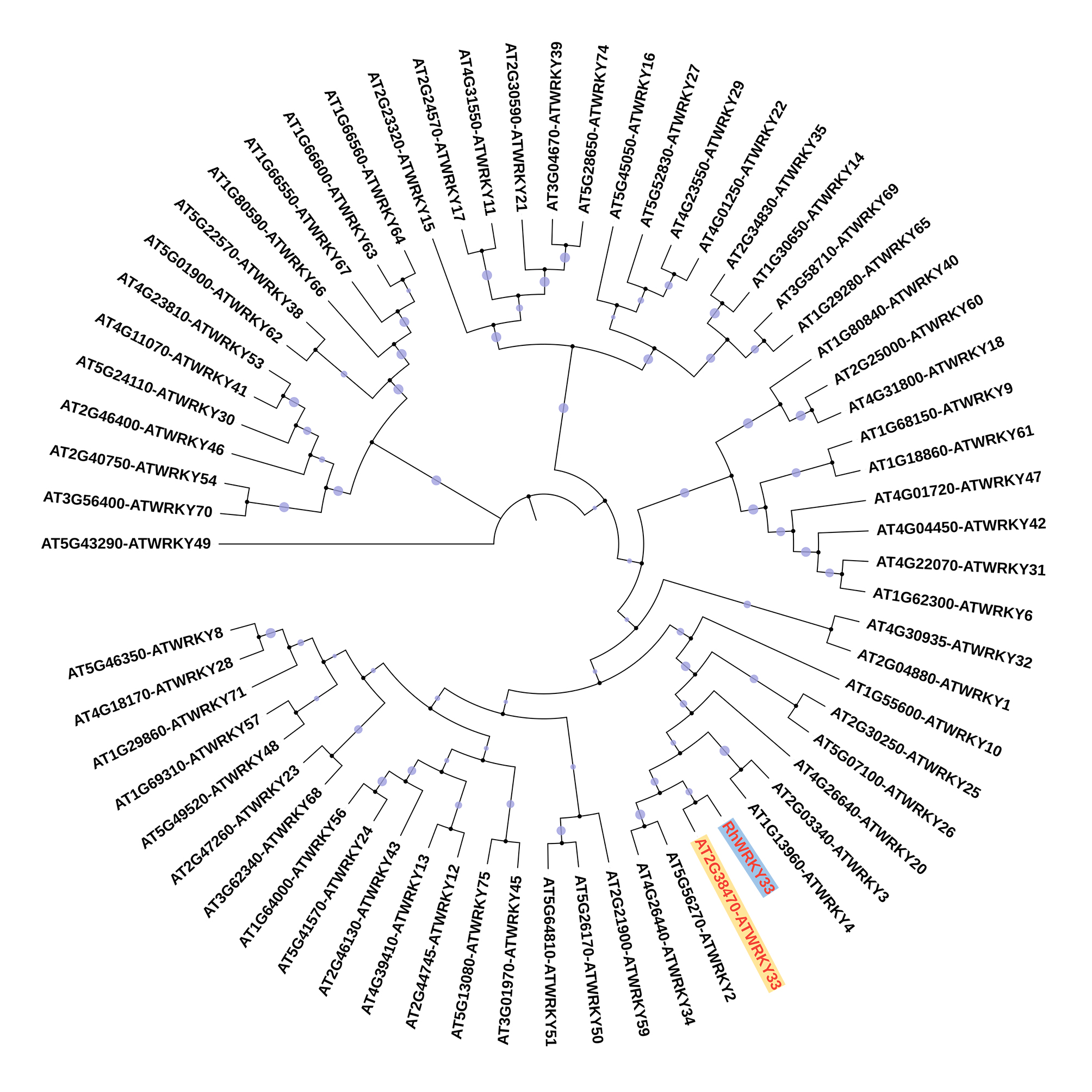

Supplement: Supplementary Figure 3 — Biochemical characteristics and spatiotemporal expression analysis of RhWRKY33. [file Image_3.JPEG]

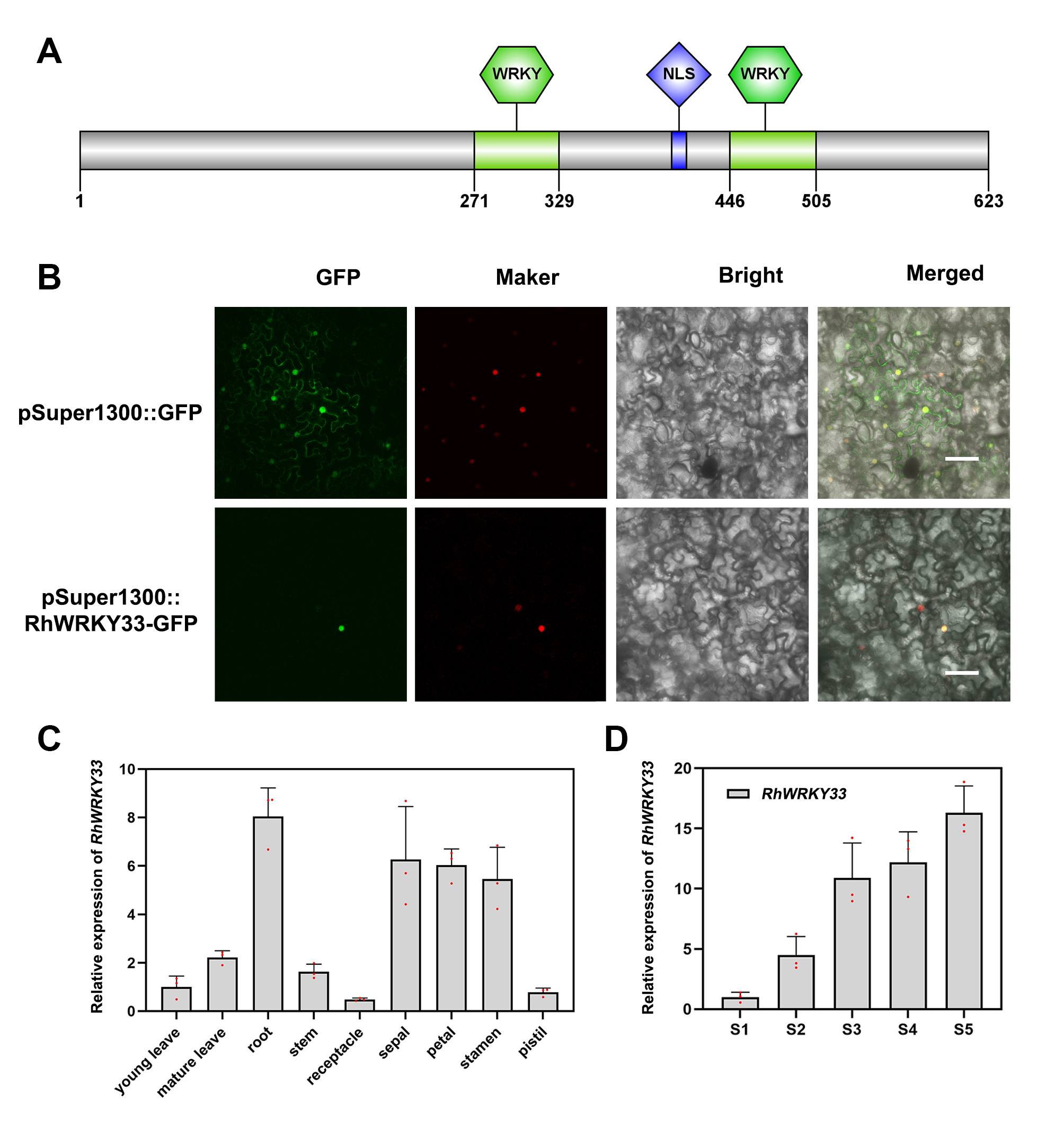

Supplement: Supplementary Figure 4 — Display of DEGs relating to transcription factors, protein modification, protein degradation (A), and hormone signaling (B) was specifically regulated by Group 3. [file Image_4.JPEG]

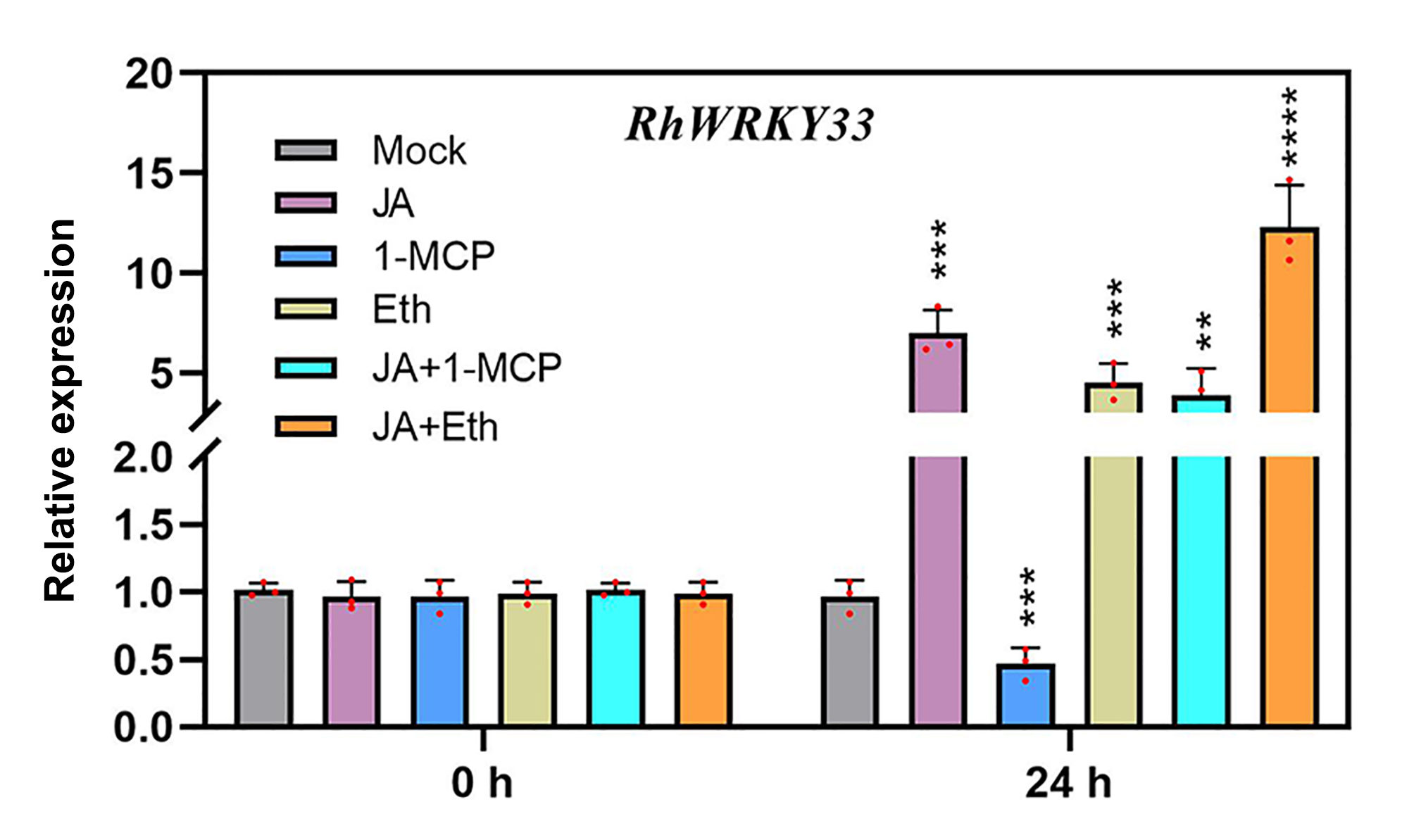

Supplement: Supplementary Figure 5 — Effects of different hormone treatments on the expression of RhWRKY33. [file Image_5.JPEG]
